# Screen-Printed Soft-Nitrided Carbon Electrodes for Detection of Hydrogen Peroxide

**DOI:** 10.3390/s19173741

**Published:** 2019-08-29

**Authors:** Chidiebere I. Ogbu, Xu Feng, Samson N. Dada, Gregory W. Bishop

**Affiliations:** 1 Department of Chemistry, East Tennessee State University, Johnson City, TN 37614, USA; 2 Surface Analysis Laboratory, Department of Chemistry, Virginia Polytechnic Institute and State University, Blacksburg, VA 24061, USA

**Keywords:** screen-printed electrode, amperometric sensor, nitrogen-doped carbon, soft nitriding

## Abstract

Nitrogen-doped carbon materials have garnered much interest due to their electrocatalytic activity towards important reactions such as the reduction of hydrogen peroxide. N-doped carbon materials are typically prepared and deposited on solid conductive supports, which can sometimes involve time-consuming, complex, and/or costly procedures. Here, nitrogen-doped screen-printed carbon electrodes (N-SPCEs) were fabricated directly from a lab-formulated ink composed of graphite that was modified with surface nitrogen groups by a simple soft nitriding technique. N-SPCEs prepared from inexpensive starting materials (graphite powder and urea) demonstrated good electrocatalytic activity towards hydrogen peroxide reduction. Amperometric detection of H_2_O_2_ using N-SPCEs with an applied potential of −0.4 V (vs. Ag/AgCl) exhibited good reproducibility and stability as well as a reasonable limit of detection (2.5 µM) and wide linear range (0.020 to 5.3 mM).

## 1. Introduction

Over the past few decades, screen-printed carbon electrodes (SPCEs) have emerged as important analytical devices, especially for sensing and biosensing, due to their relatively low cost, ease of fabrication, versatility, and wide commercial availability [1,2,3]. However, the use of SPCEs as sensing and biosensing platforms usually requires surface treatment (e.g., mechanical polishing [3], UV–ozone [4], plasma [5], electrochemical activation [6,7], etc.) or modification of the electrode surface with catalysts (e.g., nanomaterials [8,9,10,11,12], enzymes [1,13,14], etc.) in order to impart adequate sensitivity and/or selectivity for electrochemical measurement of the analyte. For example, hydrogen peroxide, an important compound involved in many enzymatic reactions, including as a product of the oxidation of glucose by glucose oxidase [15], has been detected using SPCEs modified with platinum nanoparticles [8,12], carbon-based nanomaterials [9,11], various nanocomposites [16,17], surfactants [18], Prussian blue [10,19], and enzymes [1,13,14], as well as surface treatment using oxygen plasma [5] and electrochemical activation [7]. While these strategies have facilitated electrochemical measurement of H_2_O_2_ at low limits of detection and with high sensitivity, wider adoption of these techniques outside of the research lab can often be hampered by time-consuming or complex protocols and high costs associated with the required materials and processing equipment.

Recently, nitrogen-doped carbon materials have found increasing use in H_2_O_2_ sensing. N-doped carbon materials can be produced from various methods such as pyrolysis of iron(II) phthalocyanine [20,21] or pyridine/ferrocene mixtures [22,23], nitrogen plasma treatment of graphene [24,25] or carbon nanofibers [26], hydrothermal treatment of carbon nanofiber powder with urea [26], thermal annealing of graphene oxide with ammonia [27], and heating carbon nanotubes or graphene nanoribbons in the presence of ammonium hydroxide [11]. The doping of nitrogen atoms into graphitic structures introduces free electrons and facilitates O–O bond breakage at these electrocatalytically active sites [11]. Since many strategies for preparing N-doped carbon materials and nanomaterials that are commonly used in N-doping processes (e.g., carbon nanotubes, graphene oxide, etc.) involve metal-containing precursors (e.g., iron(II) phthalocyanine) or oxidizing agents (e.g., permanganate), the possible presence of metal impurities and their effects on electrocatalytic performance of N-doped carbon materials have also been scrutinized [28,29,30,31,32]. For N-doped carbon preparation strategies involving metals, great efforts have been made to demonstrate complete removal of metal impurities [21,28] or document their inability to behave electrocatalytically due to their encapsulation within N-doped carbon structures [20]. However, the desire to eliminate the possible introduction of metal impurities and the subsequent need for their removal has spurred much research in the development of metal-free protocols for preparing N-doped carbon materials [28,29,30,31,32]. 

Recently, Liu et al. reported that nitrogen groups can be introduced onto various carbon blacks, mesoporous carbons, and activated carbons through simple low-temperature annealing with urea (soft nitriding) [33]. Thermolysis of urea is a metal-free process that produces isocyanic acid and ammonia, which are thought to react mostly with oxygenated surface sites on carbon materials, resulting in the incorporation of ureido groups and ketimine functionalities, respectively. While N-doping of carbon materials through soft nitriding was found to enable immobilization of highly electrocatalytically active metal nanoparticles through reduction of metal precursor ions via sodium borohydride [33,34], the electrocatalytic behavior of the soft-nitrided carbon materials themselves towards hydrogen peroxide reduction has not yet been reported. 

In these studies, we show that SPCEs prepared from nitrogen-doped graphite (N-SPCEs) are suitable, economical platforms for electrocatalytic reduction of H_2_O_2_. N-SPCEs are produced from low-cost starting materials (graphite and urea) by soft nitriding and the use of screen-printing eliminates the need to distribute N-doped carbon on other solid electrode supports. N-SPCEs are applied for amperometric detection of H_2_O_2_ and exhibit sensitivity, reproducibility, and long-term stability that are comparable to other H_2_O_2_ sensors based on N-doped carbon materials supported on SPCEs or glassy carbon electrodes (GCEs).

## 2. Experimental

### 2.1. Materials

Graphite powder, urea, cellulose acetate, acetone, cyclohexanone, potassium chloride, ferrocenemethanol (FcMeOH), potassium ferricyanide, potassium ferrocyanide, bovine calf serum, 2,2′-Azino-bis (3-ethylbenzothiazoline-6-sulfonic acid) diammonium salt (ABTS), and horseradish peroxidase (HRP, type II, 200 units/mg) were obtained from Sigma-Aldrich. Hydrogen peroxide (30 wt %), sodium phosphate dibasic and potassium phosphate monobasic were purchased from Acros Organics. Phosphate buffered saline (PBS) tablets were obtained from Fisher Scientific, and perchloric acid (70%) was from Fluka. All aqueous solutions were prepared with 18.2 MΩ cm ultrapure water obtained by passing deionized water through a Millipore Synergy purifier.

### 2.2. Preparation of N-Doped Graphite

Nitrogen doping of graphite was achieved through a previously reported metal-free soft nitriding protocol [33] with slight modification. Graphite powder (1 g) was mixed with solid urea (1.5 g) and annealed in an oven at 150 °C for two hours followed by 250 °C for two hours. The product was washed with water and ethanol three times. The resulting N-doped graphite was then collected and dried at 70 °C before use.

### 2.3. Characterization of Carbon Materials

Scanning electron microscopy (SEM) was performed using an FEI Quanta 600 FEG environmental scanning electron microscope and a Bruker QUANTAX 400 energy dispersive X-ray spectrometer (EDS). X-ray photoelectron spectroscopy (XPS) analyses of graphite and N-doped graphite were carried out using a PHI VersaProbe III scanning XPS microscope equipped with a monochromatic Al K-α X-ray source (1486.6 eV). Spectra were acquired with 200 µm/50 W/15 kV X-ray settings and dual-beam charge neutralization. Binding energies were referenced to sp^2^ carbon peak at 284.3 eV. Atomic surface concentrations (%) of elements were determined from the integrated intensity of the elemental photoemission features corrected by relative atomic sensitivity factors. Fourier-transform infrared spectroscopy (FTIR) was performed using a Shimadzu IRPrestige-21 spectrometer equipped with a Pike MIRacle ATR sampling accessory. Spectra were obtained from the average of 16 scans from 600 to 4000 cm^−1^ at 4 cm^−1^ resolution.

### 2.4. Preparation of SPCEs and N-SPCEs

Simple conductive inks composed of graphite or N-doped graphite in a cyclohexanone–acetone solvent mixture (1:1 by volume) with cellulose acetate as the polymeric binder were prepared based on previous reports [35,36]. Briefly, cellulose acetate (0.06 g), cyclohexanone (1 mL), and acetone (1 mL) were mixed together and sonicated for 20 min to obtain a homogenous mixture. Then, graphite or N-doped graphite powder (0.94 g) was added, and the mixture was sonicated for an additional 40 min. SPCE and N-SPCE working electrodes were manually printed onto plastic cellulose acetate sheets using a 110-mesh screen prepared as previously described [37].

### 2.5. Electrochemical Measurements

Cyclic voltammetry (CV) and amperometry were performed using a CH Instruments 1040C electrochemical analyzer with a SPCE or N-SPCE working electrode, Ag/AgCl (1 M KCl) reference electrode (CH Instruments), and a platinum wire counter electrode. All currents were converted to current density by normalizing measured current by the geometric surface area of the working electrode (0.028 cm^2^ [37]) [11,26,38,39]. Chronocoulometry and electrochemical impedance spectroscopy were carried out using a CH Instruments 760E electrochemical analyzer. Chronocoulometry of 0.5 mM FcMeOH in 0.1 M KCl was performed by stepping the potential of the working electrode (SPCE or N-SPCE) from 0 V to 100 mV more positive than peak potential associated with oxidation of FcMeOH to FcMeOH^+^ based on CV experiments performed at a scan rate of 50 mV s^−1^. EIS measurements were recorded in 5 mM Fe(CN)_6_^3−/4−^ in 0.1 M KCl at the open-circuit potential (+0.22 V vs. Ag/AgCl) using a frequency range of 10 kHz–0.1 Hz and AC perturbation amplitude of 5 mV. EIS data were fitted to an equivalent circuit model using EIS Analyser Software [40].

### 2.6. Spectrophotometric Assay for H_2_O_2_

A previously reported spectrophotometric H_2_O_2_ assay based on ABTS-HRP [41] was carried out for comparison to electrochemical detection. Briefly, 1 mL of a solution containing 0.0144 mM ABTS and 0.021 mg mL^−1^ HRP was added to a cuvette. Thirty microliters of H_2_O_2_ standard (prepared in 0.1 M HClO_4_) or sample was introduced and quickly mixed in the cuvette containing the ABTS-HRP solution. The absorbance was measured at 414 nm using a UV-Vis spectrophotometer (Shimadzu UV-1700 Pharmaspec). The reference for UV-Vis measurements consisted of 1 mL of the ABTS-HRP solution mixed with 30 µL of 0.1 M HClO_4_ as previously described [41].

## 3. Results

### 3.1. Characterization of Nitrogen-Doped Graphite

**Figure 1:**
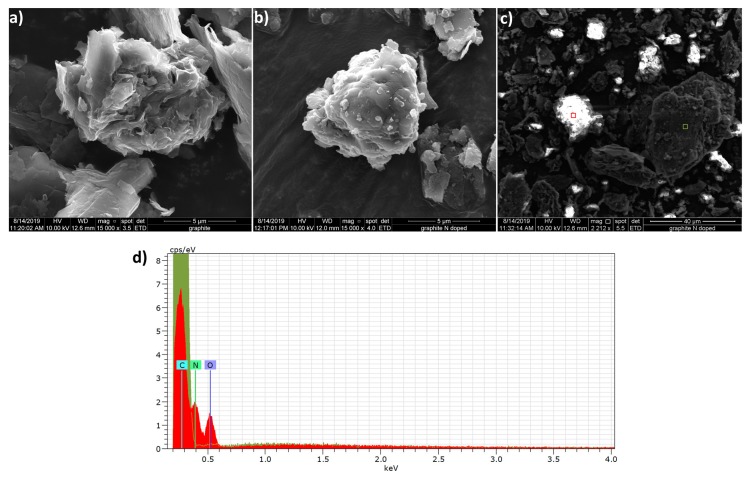
SEM/EDS of graphite and N-doped graphite. SEM images depict untreated (**a**) and N-doped graphite (**b**) particles. An SEM image of N-doped graphite obtained without prior application of Au/Pd conductive coating (**c**) exhibits areas of high surface charging (bright spots). Red and green boxes correspond to areas analyzed by EDS. EDS analysis (**d**) shows the absence of oxygen and nitrogen in an area of low surface charging (green shaded spectrum corresponding to the green box in (**c**) and the presence of these atoms in an area of high surface charging (red shaded spectrum corresponding to the red box in (**c**)).

Soft nitriding did not appear to significantly change the overall surface morphology/structure of graphite particles based on SEM images (Figure 1). However, N-doped graphite particles did seem to have smoother and more rounded edges (Figure 1b) than untreated graphite (Figure 1a). N-doped graphite samples imaged without prior application of an Au/Pd coating exhibited areas of intense surface charging (Figure 1c), which were not observed on untreated graphite samples and are indicative of the presence of insulating surface atoms such as nitrogen and oxygen. EDS analysis of high surface charging areas confirms the existence of nitrogen and oxygen atoms on the N-doped graphite surface (Figure 1d).

**Figure 2:**
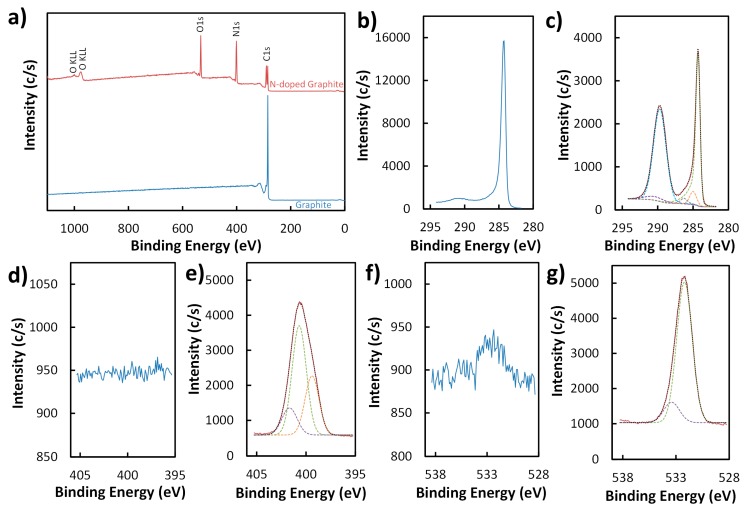
XPS survey (**a**) and high-resolution (**b**–**g**) spectra of graphite (blue) and N-doped graphite (red). High-resolution spectra display the C1s (**b**,**c**), N1s (**d**,**e**), and O1s (**f**,**g**) regions for graphite (**b**,**d**,**f**) and N-doped graphite (**c**,**e**,**g**). Models (black dotted lines) of C1s (**c**), N1s (**e**), and O1s (**g**) peaks for N-doped graphite (solid red lines) based on deconvolution of the peaks into their major components (green, purple, orange, gray, and light blue dashed lines) are also shown.

XPS of untreated graphite exhibited the characteristic intense C1s peak at 284.3 eV (Figure 2a,b) corresponding to sp^2^ carbon atoms and a broad peak at ~291 eV (Figure 2b) consistent with the π-π* shake-up transition of graphite as expected [42,43]. A very weak O1s peak at ~532 eV (0.2%) was also present, presumably from air contamination (Figure 2f). After low-temperature annealing with urea, XPS survey of the resulting N-doped graphite displayed the notable addition of N1s and O1s signals (Figure 2a). Relative atomic surface concentrations for nitrogen (32.9%) and oxygen (19.1%) on N-doped graphite indicate that a significant amount of nitrogen/oxygen-containing groups are introduced on the graphite surface by soft nitriding.

Thermolysis of urea is known to produce isocyanic acid and ammonia, which reportedly introduce ureido groups and n-doping on graphitic layers, respectively, primarily at oxygen-containing sites of carbon materials [33]. However, graphite contains a much lower proportion of such sites—only 0.2% relative surface atomic O concentration was measured by XPS in this work (Figure 2a,f)—compared to Printex G carbon black (16% relative surface atomic O concentration according to a previous study [33]). Besides isocyanic acid and ammonia, thermal decomposition of urea is known to also lead to the formation of various other products through condensation and polymerization reactions [44,45,46,47]. Schaber et al. found that pyrolysis of urea in an open reaction vessel at 250–275 °C resulted primarily in cyanuric acid, ammelide, and ammeline with small amounts of melamine and biuret [45]. Studies have also shown that urea [48] and 1,3,5-triazines [42,49] (e.g., cyanuric acid, ammelide, ammeline, and melamine) can strongly adsorb on graphite and even intercalate between graphene layers. 1,3,5-triazines with –NH_2_ and –OH substituents can form highly stable 2D networks and supramolecular aggregates [50,51,52,53,54] and can also interact strongly with graphite surfaces through hydrogen bonding [49]. 

The N1s (Figure 2e) and C1s (Figure 2c) features of N-doped graphite appear to support the idea that heating urea in the presence of graphite leads to modification of the carbon surface with 1,3,5-triazines. The N1s feature can be deconvoluted into three peaks (Figure 2e) at 399.4 eV, 400.7 eV, and 401.7 eV. The peak at 399.4 eV can be indexed to pyridinic C-N=C of 1,3,5-triazines [52,55]. The largest peak at 400.7 eV is consistent with amide N of keto tautomers of alcohol-substituted 1,3,5-triazines (i.e., cyanuric acid, ammelide, ammeline) [55]. The trione form of cyanuric acid (isocyanuric acid) is known to be favored [55,56,57], which helps explain the larger surface atomic concentration of amide N (55.6%) compared to pyridinic N (30.7%). The smallest peak at 401.7 eV corresponds to amine N atoms in amine-substituted 1,3,5-triazines (i.e., ammelide, ammeline, melamine) [52,55]. In addition to characteristic graphitic carbon signatures, three more peaks at 285.0 eV, 286.3 eV, and 289.7 eV are deconvoluted from the C1s feature of the N-doped graphite sample (Figure 2c). The C1s peak at 289.7 eV provides additional evidence of surface modification as it is consistent with the presence of O=C–N groups [55]. The minor components at 285.0 eV and 286.3 eV are assigned to sp^3^ C atoms [43] and C–O or C–N [42,46,58,59], respectively. The O1s feature of N-doped graphite can be deconvoluted into two peaks (Figure 2g) at 532.2 eV and 533.4 eV, which are attributed to C=O and C–O, respectively.

**Figure 3:**
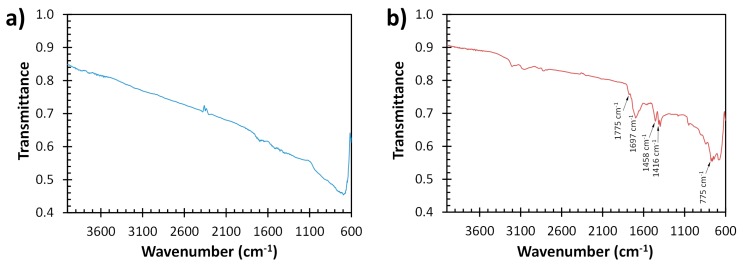
FTIR spectra of graphite (**a**) and N-doped graphite (**b**).

The FTIR spectrum of N-doped graphite exhibits several bands, which are not observed in the spectrum of untreated graphite, and can be attributed to the presence of 1,3,5-triazine compounds (Figure 3). A broad peak centered at 1697 cm^−1^ in the FTIR spectrum of N-doped graphite (Figure 3b) is consistent with characteristic C=O stretching of cyanuric acid [52]. Broad peaks at 3206 cm^−1^ and 3055 cm^−1^ are attributed to N–H stretching [60], and strong peaks at 1458 cm^−1^ and 1416 cm^−1^ are attributed to stretching modes in C–N heterocycles [61]. The small peak at 1775 cm^−1^ and peak at 775 cm^−1^ may indicate hydrogen bonding between 1,3,5-triazines as previous studies found that such interactions encountered during the formation of supramolecular aggregates of cyanuric acid and melamine shifted the C=O stretching vibration of cyanuric acid and triazine ring vibration of melamine, respectively, to these regions of the IR spectrum [52,53].

### 3.2. Electrochemical Characterization and Electrocatalytic Behavior of SPCEs and N-SPCEs

**Figure 4:**
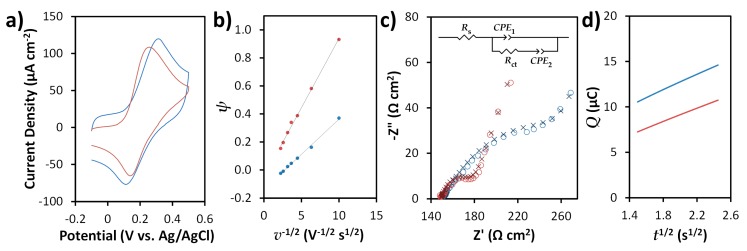
Electrochemical characterization of screen-printed carbon electrodes (SPCEs) (blue) and N-doped screen-printed carbon electrodes (N-SPCEs) (red). (**a**) Representative CVs for 0.5 mM FcMeOH with 0.1 M KCl obtained at a scan rate of 50 mV s^−1^. (**b**) Plots for determining heterogeneous electron transfer rate constant *k*^0^ for the FcMeOH/FcMeOH^+^ redox reaction based on the relationship between ∆*E*_P_ (expressed in terms of dimensionless kinetic parameter *ψ*) and scan rate *v*. (**c**) Nyquist plots (◦) and models of data (x) based on an equivalent circuit (inset) for EIS measurements in 5 mM Fe(CN)_6_^3−/4−^ in 0.1 M KCl. (**d**) Anson plot used to determine electroactive surface areas from oxidation of 0.5 mM FcMeOH in 0.1 M KCl.

Electrochemical performance of SPCEs is known to depend on factors like the structural characteristics of carbon (e.g., edge plane-like sites/defects, etc.) used in the ink formulation as well as the ratio and distribution of insulating polymeric and conductive domains in the printed electrode [62,63,64]. SPCEs and N-SPCEs prepared from graphite- and N-doped graphite-based inks, respectively, exhibited similar electrochemical behavior towards the FcMeOH/FcMeOH^+^ redox couple in cyclic voltammetry (CV) experiments in terms of peak current density (Figure 4a). However, smaller peak separation (109 mV vs. 201 mV with 50 mV s^−1^ scan rate) and lower charging current were observed using N-SPCEs. Though peak separations (∆*E*_P_) for the FcMeOH/FcMeOH^+^ redox couple on both SPCEs and N-SPCEs are much larger than the expected Nernstian value of 59 mV, the results are comparable to commercially available SPCEs and SPCEs prepared from commercially available inks [9,37,63]. 

∆*E*_P_ is known to be related to electron transfer kinetics, and can be used to estimate the heterogeneous electron transfer rate constant *k*^0^, which often serves as a basis for evaluating the performance of electrode materials [37,63,65,66]. For a quasireversible system, ∆*E*_P_ is a function of voltammetric scan rate (*v*). The link between ∆*E*_P_, *v*, and *k*^0^ is made through *ψ*, a dimensionless kinetic parameter introduced by Nicholson [67,68] and related to ∆*E*_P_ (in mV) through an empirical equation reported by Lavagnini et al. [69]:

*ψ* = [−0.6288 + 0.0021(∆*E*_P_)]/[1 − 0.017(∆*E*_P_)] (1)

The relationship between *ψ* and *k*^0^ is given by:

*ψ* = *k*^0^[*πDnFv*/(*RT*)]^−1/2^ (2)

where *D* is the diffusion coefficient of the electroactive species (*D* = 7.80 × 10^−6^ cm^2^ s^−1^ [37] for FcMeOH), *n* is the number of electrons involved in the Faradaic reaction, *F* is the Faraday constant, *R* is the gas constant, and *T* is temperature. To determine *k*^0^, Equation (1) is applied to calculate *ψ* for ∆*E*_P_ obtained at different *v*, and *ψ* is plotted as a function of *v*^−1/2^. The slope of linear plot is related to *k*^0^ by Equation (2). ∆*E*_P_ values for the FcMeOH/FcMeOH^+^ redox couple using both SPCEs and N-SPCEs were found to vary with *v* over the range of 10 to 200 mV s^−1^, and plots of *ψ* as a function of *v*^−1/2^ (Figure 4b) displayed excellent linearity (R^2^ > 0.996). Based on slopes of the linear fits of *ψ* vs. *v*^−1/2^ and Equation (2), *k*^0^ values for FcMeOH/FcMeOH^+^ were found to be 1.56 × 10^−3^ cm s^−1^ and 3.07 × 10^−3^ cm s^−1^ with SPCE and N-SPCE, respectively. These results indicate improved electron transfer at N-SPCEs compared to SPCEs by about two-fold, and are in agreement with those reported for other SPCEs using FcMeOH/FcMeOH^+^ and other common redox probes [37,63,65].

Electrochemical performance of SPCEs and N-SPCEs was also characterized by EIS using the Fe(CN)_6_^3−/4−^ redox system (Figure 4c). The experimental data was fitted to an equivalent circuit model (Figure 4c, inset), which was previously utilized by Randviir to model impedance of various redox probe systems, including ascorbic acid (which has been classified by Chen & McCreery as a probe similar to Fe(CN)_6_^3−/4−^ due to its sensitivity to carbon electrode surface characteristics [70]) on SPCEs prepared from commercially available inks [66]. *R*_s_ represents the resistance of the electrolyte solution [66]. *R*_ct_ corresponds to the charge transfer resistance, and *CPE*_1_ and *CPE*_2_ are constant phase elements used to model nonideal capacitive behavior due to surface heterogeneity [71]. Compared to SPCE, N-SPCE exhibited lower *R*_ct_ (32 (± 1.2) Ω cm^2^ vs. 158 (± 4.2) Ω cm^2^) for Fe(CN)_6_^3−/4−^ based on the fitted EIS data, which is indicative of improved electron transfer kinetics and related to *k*^0^ by [65,66]:

*k*^0^ = *RT*/(*n*^2^*F*^2^*R*_ct_*AC*) (3)

where *R*, *T*, *n*, and *F* have the same meanings as in Equation (2), and *A* and *C* correspond to the area of the electrode and concentration of the electroactive species, respectively. Based on *R*_ct_ determined from EIS and Equation (3), *k*^0^ for Fe(CN)_6_^3−/4−^ is 3.33 (± 0.088) × 10^−4^ cm s^−1^ using SPCE and 1.67 (± 0.060) × 10^−3^ cm s^−1^ using N-SPCE. Previous studies have reported *k*^0^ for Fe(CN)_6_^3−/4−^ that range from 1.67 × 10^−5^ cm s^−1^ to 8.2 × 10^−3^ cm s^−1^ with commercially available SPCEs and SPCEs prepared from commercially available inks [6,63,72,73]. 

The electroactive areas of SPCEs and N-SPCEs were estimated from chronocoulometric measurements according to the Anson equation [11,74,75]:

*Q* = *Q_dl_* + *Q_ads_* + 2*nFA*_e_*C*(*Dt*/*π*)^1/2^ (4)

where *Q_dl_* and *Q_ads_* are the charges associated with double-layer charging and Faradaic reactions of adsorbed species, respectively, *A*_e_ is electroactive area, *t* is time, and *n*, *F*, *C*, and *D* have the same meanings as in Equations (2) and (3). Plots of *Q* vs. *t*^1/2^ (Figure 4d) for the chronocoulometric oxidation of FcMeOH showed excellent linear behavior in the expected time range [75] with slopes that are related to *A*_e_ by Equation 4. Based on this method, the electroactive areas of SPCEs and N-SPCEs are 0.028 (±0.0036) cm^2^ and 0.024 (±0.0029) cm^2^ (*n* = 3), respectively, which are consistent with the geometric area (0.028 cm^2^) of the electrodes.

**Figure 5:**
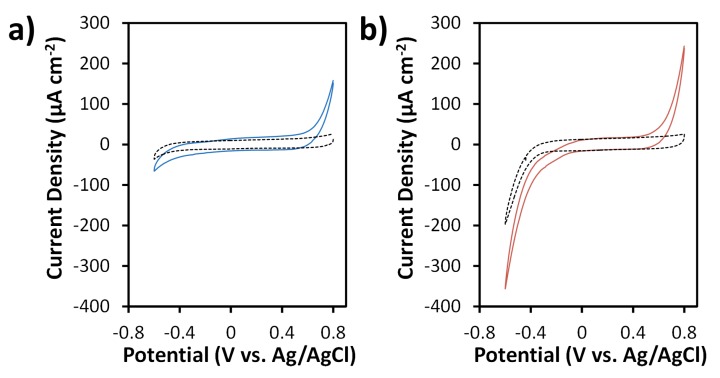
CVs of SPCE (**a**) and N-SPCE (**b**) in the absence (dashed lines) and presence (solid lines) of 20 mM H_2_O_2_ in 0.05 M phosphate buffer (pH 7.4). All CVs were obtained at a scan rate of 50 mV s^−1^.

CV experiments were carried out in the absence and presence of 20 mM H_2_O_2_ in 0.05 M phosphate buffer (pH 7.4) in order to evaluate reduction of H_2_O_2_ on SPCEs and N-SPCEs (Figure 5). Compared to SPCEs (Figure 5a), N-SPCEs exhibited a positive shift in potential for the onset of the H_2_O_2_ reduction reaction by ~150 mV and an increase in reduction current density at potentials more negative than −0.2 V (Figure 5b), indicative of electrocatalytic behavior of N-SPCEs towards H_2_O_2_ reduction.

### 3.3. Amperometric Detection of H_2_O_2_ Using N-SPCEs

**Figure 6:**
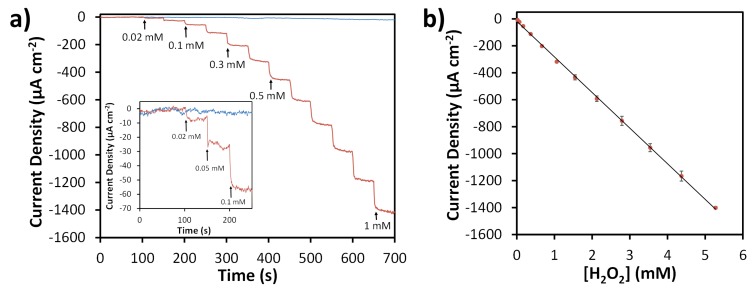
Amperometric detection of H_2_O_2_ in 0.05 M phosphate buffer (pH 7.4) at −0.4 V vs. Ag/AgCl. Representative current-time traces obtained from consecutive injections of H_2_O_2_ (**a**) using SPCE (blue) and N-SPCE (red). Arrows and labels indicate spike times and concentrations. Inset shows low concentration injections. Calibration curve (**b**) obtained from average results of four N-SPCEs. Error bars correspond to ±1 standard deviation.

Amperometric responses of both SPCEs and N-SPCEs towards H_2_O_2_ using an applied potential of −0.4 V were evaluated by successively injecting varying concentrations of H_2_O_2_ into a stirred solution of 0.05 M phosphate buffer (pH 7.4) (Figure 6). SPCEs showed no significant change in measured current density upon addition of H_2_O_2_, whereas N-SPCEs exhibited a rapid increase in reduction current density, reaching steady-state in <10 s when H_2_O_2_ was injected into the solution. The relationship between current density and concentration for H_2_O_2_ sensing using N-SPCEs showed excellent linearity (R^2^ = 0.9995) and good reproducibility (<5.5% relative standard deviation for average responses of four different N-SPCEs) in the range of 0.02 to 5.3 mM H_2_O_2_ (Figure 6b). The sensitivity of N-SPCE sensors for H_2_O_2_ was found to be 264 (± 5.3) µA mM^−1^ cm^−2^ based on the slopes of calibration curves for four different N-SPCEs. The limit of detection (LOD) calculated based on three times the standard deviation of the background signal was 2.5 µM. Sensor stability was evaluated by making repeated measurements of 1 mM H_2_O_2_ using a N-SPCE over a period of 14 days, while storing the N-SPCE at room temperature between measurements. The N-SPCE retained 98% of its initial response towards H_2_O_2_ reduction after two days, 92% after one week, and 89% after two weeks, which is comparable to the reported durability of other H_2_O_2_ sensors based on N-doped carbon materials [11,76]. 

**Figure 7:**
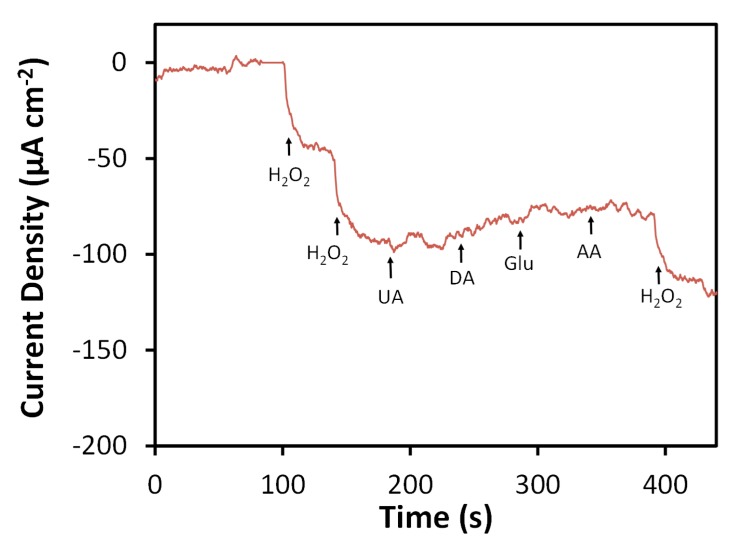
Amperometric response of N-SPCE towards H_2_O_2_ and common interfering species in 0.05 M phosphate buffer (pH 7.4) at −0.4 V vs. Ag/AgCl. Arrows and labels indicate injection times and species. Spiked concentrations for H_2_O_2_, uric acid (UA), dopamine (DA), glucose (Glu), and ascorbic acid (AA) were 0.1 mM for each injection.

Selectivity of the N-SPCE for H_2_O_2_ detection was evaluated by measuring amperometric responses towards species commonly reported to interfere with detection of H_2_O_2_ (e.g., uric acid (UA), dopamine (DA), glucose (Glu), ascorbic acid (AA) [11,26,38,39,77,78]) (Figure 7). At an applied potential of −0.4 V vs. Ag/AgCl, the N-SPCE showed no significant response towards UA, DA, Glu, or AA when these species were spiked into 0.05 M phosphate buffer (pH 7.4) to concentrations of 0.1 mM. Performance of the N-SPCE-based H_2_O_2_ sensor in a complex matrix was assessed through measurement of H_2_O_2_ in bovine calf serum, which has been reported to be a suitable and convenient alternative to human serum for use in the development electrochemical biosensors [79]. Bovine serum was mixed with H_2_O_2_, and 6 to 500 µL of the mixture was injected into 20 mL of 0.05 M phosphate buffer so that the spiked H_2_O_2_ concentration could be determined based on the response measured at the N-SPCE using an applied potential of −0.4 V. Measured concentrations were in good agreement with those obtained separately by a previously reported spectrometric assay based on the ABTS-HRP system [41] (Table 1). However, amperometric detection of H_2_O_2_ using N-SPCEs offers a simpler procedure and larger linear range, whereas spectrometric analysis of spiked samples containing higher concentrations required prior dilution due to the more limited linear range of the method (10 to 200 µM). Average recoveries for amperometric detection of H_2_O_2_ using N-SPCEs ranged from 91 to 100% for spike concentrations of 0.015 to 2.50 mM. These results are comparable to those obtained from similar experiments for detection of H_2_O_2_ in the presence of dilute human serum using SPCEs modified with a reduced graphene oxide-persimmon tannin-platinum nanoparticle composite [17] and GCEs modified with nitrogen-doped carbon nanoparticles embedded in a carbon nanofiber film [38].

## 4. Discussion

Low-temperature annealing of carbon blacks, activated carbons, and mesoporous carbons with urea was recently reported as a method for introducing nitrogen groups (soft nitriding) onto the surfaces of these materials [33]. Nitrogen atoms are believed to be incorporated primarily through reactions of isocyanic acid and ammonia with oxygenated surface sites. Based on the apparent importance of surface oxygen species, the lack of oxygen-containing sites would seem to impede the success of soft nitriding of graphite. However, in studies presented here, annealing of graphite with urea at 250 °C similarly introduced nitrogen groups, with XPS and FTIR indicating the presence of pyridinic, amide, and amine N as well as CN heterocycles consistent with formation of 1,3,5-triazines. 1,3,5-triazines are known to result from the thermal decomposition of urea [44,45,46,47], interact strongly with graphene layers through hydrogen bonding [42], and form supramolecular aggregates [50,51,52,53,54]. Application of a temperature high enough to produce 1,3,5-triazines from urea but low enough to avoid sublimation/decomposition of these compounds, which reportedly begins at temperatures >250 °C [45], is likely crucial to achieve successful modification as graphite contains few surface oxygenated species. A previous study reported that thermal annealing of graphite in the presence of urea at 600 °C resulted in a surface atomic N concentration of only 1.11% by XPS [80], while we found annealing at 250 °C led to 32.9%. A relatively large percentage (30.7%) of these N atoms were indexed to pyridinic N, which have been linked to electrocatalytic activity towards H_2_O_2_ reduction [31,32,39,78,80].

Graphite-like conjugated aromatic CN supramolecular assemblies are known to develop from polycondensation of 1,3,5-triazines formed upon heating urea and can even lead to production graphitic carbon nitride (g-C_3_N_4_) if urea is heated to a temperature of 450–550 °C [46]. Though polycondensation of 1,3,5-triazines directly from thermal treatment of urea reportedly requires temperatures of 400 °C or higher [46], a previous study showed that polycondensation of melamine and cyanuric acid produced directly from urea can proceed at temperatures as low as 250 °C when urea is heated in the presence of tetraethylorthosilicate [47]. Thermal annealing of graphite with urea at 250 °C may have also produced some graphite-like conjugated aromatic CN supramolecular assemblies of 1,3,5-triazines in these studies, which could help explain the large surface nitrogen content found in N-doped graphite by XPS despite the lack of oxygen-containing surface sites in the graphite starting material. While there are several methods to introduce oxygenated surface sites on graphite, these involve additional processing and purification steps, may require additional equipment or instrumentation, and can sometimes introduce impurities that can affect electrocatalytic properties [32]. 

N-SPCEs prepared from N-doped graphite ink were not significantly different from SPCEs in terms of electroactive area. However, they did exhibit a lower *R*_ct_ for Fe(CN)_6_^3−/4−^ and a larger *k*^0^ for FcMeOH/FcMeOH^+^ than SPCEs, which are indicative of improved electrochemical performance. Enhancement of electron transfer kinetics by incorporation of nitrogen in carbon materials was previously documented in comparative studies of amorphous carbon thin film electrodes (a-C) and nitrogenated a-C (a-C:N) [81,82,83]. Yang et al. found that *k*^0^ for the Fe(CN)_6_^3−/4−^ redox probe system increased from 5 × 10^−3^ cm s^−1^ to 1.5 × 10^−2^ cm s^−1^ with increasing flow rate (0–50 sccm) of N_2_ used during preparation of a-C and a-C:N via pulsed laser-arc deposition of carbon [81]. The reason activity towards Fe(CN)_6_^3−/4−^ was greater for a-C:N compared to a-C was not clear, but could be the result of higher active site density or differences in double layer structure, which can have large impact on this surface-sensitive inner-sphere redox probe [81]. Behan et al. reported that *R*_ct_ for Ru(NH)_6_^3+/2+^, which like FcMeOH/FcMeOH^+^ is an outer-sphere redox probe system [75] relatively insensitive to surface microstructure (oxides, adsorbed monolayers, etc.) and largely controlled by electronic properties of the electrode [81,82,83], decreased by more than ten times using a-C:N (prepared by dc magnetron sputtering in the presence of 5% N_2_/Ar gas mixture) when compared to similarly fabricated a-C [83]. The decrease in *R*_ct_ was attributed to increased metallic character of a-C:N compared to a-C. In our studies, N-SPCEs showed approximately two times higher *k*^0^ for FcMeOH/FcMeOH^+^ and nearly five times lower *R*_ct_ for Fe(CN)_6_^3−/4−^ compared to SPCEs.

N-SPCEs also showed good electrocatalytic behavior towards reduction of H_2_O_2_ with stability and reproducibility that are similar to other electrodes modified with N-doped carbon materials. The sensitivity of N-SPCEs for amperometric detection of H_2_O_2_ was comparable to those of other N-doped carbon-based amperometric sensors, which have been reported as 28.7 to 2180 µA mM^−1^ cm^−2^ (Table 2). LODs for H_2_O_2_ using these sensors have also been reported to range from 90 to 0.609 µM, though values around 2.0 to 1.5 µM are most common. While the LOD for H_2_O_2_ obtained with N-SPCEs (2.5 µM) is slightly higher than that of most other N-doped carbons, previously reported sensors based on N-doped carbon have all involved immobilization of the materials on solid conductive supports (usually GCEs), whereas N-SPCEs are directly fabricated. Also, other reported N-doping strategies involve more cumbersome processing steps, more expensive starting materials, or additional equipment compared to soft nitriding and screen printing used in the preparation of N-SPCEs. Since soft nitriding of carbon blacks has been shown to enable deposition of highly electrocatalytically active metal nanoparticles on these carbon materials [33], the performance and versatility of N-SPCEs may also be improved by employing a similar method to develop metal nanoparticle-modified N-SPCE sensors.

## 5. Conclusions

N-SPCEs were prepared from N-doped graphite produced by simple, low-temperature annealing of graphite with urea (soft nitriding). The lack of oxygen-containing sites would seem to be impede the success of soft nitriding of graphite based on a previous report that found surface oxygen species to be the primary sites for introduction of N atoms on other carbon materials by soft nitriding. Nitrogen groups on N-doped graphite were identified as originating from −OH- and –NH_2_-substituted 1,3,5-triazines, which stand in slight contrast to the ureido and ketimine functionalities reportedly introduced primarily at oxygen-containing sites by soft nitriding of carbon black [33]. However, the formation of −OH- and −NH_2_-substituted 1,3,5-triazines during soft nitriding of graphite is consistent with previous reports on the thermal decomposition of urea at low temperatures [44,45,46,47]. N-SPCEs exhibited electrocatalytic activity towards the reduction of H_2_O_2_ and appropriate performance as H_2_O_2_ sensing platforms, which had not been previously reported for other N-doped carbons prepared by soft nitriding. The simplicity of the soft nitriding procedure, low-cost of the starting materials, and ease of electrode preparation are appealing attributes of N-SPCEs that make them a worthwhile addition to the growing number of N-doped carbon- and SPCE-based H_2_O_2_ sensing platforms. 

## Figures and Tables

**Table 1:** Spike recoveries for H_2_O_2_ in bovine calf serum measured using N-SPCE at −0.4 V in 0.05 M phosphate buffer (pH 7.4) and comparison of amperometric detection to an ABTS-HRP-based assay for H_2_O_2_ [41]. Results are presented as average (±1 standard deviation) (*n* = 3).

Spiked (mM)	Measured (mM)	% Recovery	ABTS-HRP Assay (mM)
0.015	0.0137 (±0.00019)	91 (±1.3)	0.016 (±0.0011)
0.025	0.024 (±0.0010)	97 (±4.0)	0.024 (±0.0012)
0.080	0.074 (±0.0021)	93 (±2.7)	0.080 (±0.0012)
0.40	0.39 (±0.026)	98 (±6.5)	0.39 (±0.012)
2.50	2.49 (±0.062)	100 (±2.5)	2.41 (±0.040)

**Table 2:** Comparison of some N-doped carbon-based amperometric sensors for H_2_O_2_.

Electrode	E (V) ^1^	Linear Range (mM)	Sensitivity ^2^ (µA mM^−1^ cm^−2^)	LOD (µM)	Ref.
N-Graphene/GCE	−0.2	10^−5^–2.8	NR ^3^	NR ^3^	[24]
N-GrNR ^4^/SPCE	−0.4	0.005–0.085 0.135–1.385	2180 640	1.72	[11]
g-C_3_N_4_ NS ^5^/GCE	−0.3	0.1–90	NR ^3^	2.0	[84]
N-Carbon/GCE	−0.3	0.1–40	NR ^3^	90	[77]
N-CNF_ht_^6^/GCE	−0.4	0.01–0.71 0.71–2.91	357 203	0.62	[26]
N-CNF_p_^7^/GCE	−0.4	0.01–0.21 0.21–2.21	257 180	1.84	[26]
OMCN ^8^/GCE	−0.19	0.004–0.04 0.04–12.4	642 ^9^ 288 ^9^	1.52	[76]
N-SEGN ^10^/GCE	−0.4	0.01–2.225	231.3	0.88	[39]
N-rGO ^11^/GCE	−0.4	0.01–4.625	57.3	0.94	[39]
N-CNF mat ^12^	−0.5	0.5–2.5	28.7 ^9^	0.609	[78]
N-CNPF ^13^/GCE	−0.4	0.005–27	383.9	1.5	[38]
N-SPCE	−0.4	0.02–5.3	264	2.5	This work

^1^ working electrode potential vs. Ag/AgCl reference, ^2^ based on geometric area of the working electrode, ^3^ not reported, ^4^ graphene nanoribbon, ^5^ graphitic carbon nitride nanosheet, ^6^ carbon nanofiber (hydrothermally N-doped), ^7^ carbon nanofiber (plasma N-doped), ^8^ ordered mesoporous carbon nitride, ^9^ based on sensitivity in mA mM^−1^ and geometric area reported in reference, ^10^ sonoelectrochemical graphene nanosheet, ^11^ reduced graphene oxide, ^12^ carbon nanofiber mat (polyacrylonitrile fiber embedded with carbon nanotubes), ^13^ carbon nanoparticles embedded in carbon nanofiber film.
